# The burden and spatial distribution of bovine African trypanosomes in small holder crop-livestock production systems in Tororo District, south-eastern Uganda

**DOI:** 10.1186/s13071-014-0603-6

**Published:** 2014-12-23

**Authors:** Dennis Muhanguzi, Kim Picozzi, Jan Hattendorf, Michael Thrusfield, John David Kabasa, Charles Waiswa, Susan Christina Welburn

**Affiliations:** Department of Biomolecular and Biolaboratory Sciences, School of Biosecurity, Biotechnical and Laboratory Sciences, College of Veterinary Medicine Animal Resources and Biosecurity, Makerere University, P.O. Box 7062, Kampala, Uganda; Division of Infection & Pathway Medicine, Centre for Infectious Diseases, School of Biomedical Sciences, College of Medicine and Veterinary Medicine, The University of Edinburgh, Chancellor’s Building, 49 Little France Crescent, Edinburgh, EH16 4SB UK; Department of Public Health and Epidemiology, Swiss Tropical Institute, Socinstrasse 57, CH-4002 Basel, Switzerland; University of Basel, Petersplatz 1, 4003 Basel, Switzerland; Royal (Dick) School of Veterinary Studies, The University of Edinburgh, Edinburgh, EH25 9RG UK; Department of Biosecurity, Ecosystems & Veterinary Public Health, School of Biosecurity, Biotechnical and Laboratory Sciences, College of Veterinary Medicine Animal Resources and Biosecurity, Makerere University, P.O. Box 7062, Kampala, Uganda; Department of Pharmacy, Clinical and Comparative Medicine, School of Veterinary Medicine and Animal Resources, College of Veterinary Medicine Animal Resources and Biosecurity, Makerere University, P.O. Box 7062, Kampala, Uganda

**Keywords:** African animal trypanosomiasis, Human african trypanosomiasis, Control, ITS1-PCR, Prevalence, Tororo district

## Abstract

**Background:**

African animal trypanosomiasis (AAT) is considered to be one of the greatest constraints to livestock production and livestock-crop integration in most African countries. South-eastern Uganda has suffered for more than two decades from outbreaks of zoonotic Human African Trypanosomiasis (HAT), adding to the burden faced by communities from AAT. There is insufficient AAT and HAT data available (in the animal reservoir) to guide and prioritize AAT control programs that has been generated using contemporary, sensitive and specific molecular techniques. This study was undertaken to evaluate the burden that AAT presents to the small-scale cattle production systems in south-eastern Uganda.

**Methods:**

Randomised cluster sampling was used to select 14% (57/401) of all cattle containing villages across Tororo District. Blood samples were taken from all cattle in the selected villages between September-December 2011; preserved on FTA cards and analysed for different trypanosomes using a suite of molecular techniques. Generalized estimating equation and Rogen-Gladen estimator models were used to calculate apparent and true prevalences of different trypanosomes while intra cluster correlations were estimated using a 1-way mixed effect analysis of variance (ANOVA) in R statistical software version 3.0.2.

**Results:**

The prevalence of all trypanosome species in cattle was 15.3% (95% CI; 12.2-19.1) while herd level trypanosome species prevalence varied greatly between 0-43%. *Trypanosoma vivax* (17.4%, 95% CI; 10.6-16.8) and *Trypanosoma brucei rhodesiense* (0.03%) were respectively, the most, and least prevalent trypanosome species identified.

**Conclusions:**

The prevalence of bovine trypanosomes in this study indicates that AAT remains a significant constraint to livestock health and livestock production. There is need to implement tsetse and trypanosomiasis control efforts across Tororo District by employing effective, cheap and sustainable tsetse and trypanosomiasis control methods that could be integrated in the control of other endemic vector borne diseases like tick-borne diseases.

## Background

African Animal Trypanosomiasis (AAT) is one of the most important vector-borne diseases of livestock in East Africa and common throughout the tsetse belts of Africa [1,2]. *Trypanosoma congolense*, *Trypanosoma vivax* and *Trypanosoma brucei* subspecies *brucei* are the most important causes of AAT mainly transmitted by tsetse flies (*Glossina ssp*.) [3]. AAT is usually chronic; characterized by loss of condition, progressive anaemia and often terminates in death if untreated [4]. *T. vivax* can also be transmitted mechanically by broad spectra of haematophagous insects and as a result, AAT caused by *T. vivax* has been recorded outside of the tsetse belts [5]. *T. brucei rhodesiense* and *T.b. brucei* are less pathogenic in cattle than *T.congolense* and *T. vivax* [5]. The persistent and long-term presence of *T. brucei rhodesiense* in cattle as a reservoir of human infection is of major public health importance [6] with spillover from domestic livestock causing human *T.b. rhodesiense* African trypanosomiasis (HAT), also known as sleeping sickness [7-9]. Domestic animals of epidemiological importance, are, notably cattle, which act as reservoirs of the human infective trypanosomes [10-12].

In Sub-Saharan Africa and south-eastern Uganda in particular, vector-borne diseases notably AAT constrain livestock production and compound poverty levels contributing to 34% of all livestock keepers to subsist on less than 1.24 USD per day [13-15]. Poor livestock health as a result of AAT denies farmers the opportunity to use draft power and manure as the gateway to crop-livestock enterprise integration thereby extending this problem of poverty and hunger in the tsetse-infested areas [1,2]. Hunger continues to affect more than one third of the populations in this region [16,17].

There is little contemporary data available on AAT burden generated from the sensitive and specific molecular techniques that are widely available. Such data is essential for guiding and prioritising tsetse control and the integration of such control efforts with those of other endemic vector-borne diseases such as tick-borne diseases (TBDs). The present study represents the first large scale attempt to generate reliable prevalence data from which to evaluate the extent to which the African trypanosomiasis presents a problem in small scale cattle production systems in south-eastern Uganda. A cross sectional survey was undertaken to determine both the prevalence and spatial distribution of African trypanosomes across Tororo District.

## Methods

### Study area

The survey was carried out in Tororo during the period September to December 2011. Tororo District is bordered by the districts of Mbale to the north, Manafwa to the north-east, Busia to the south, Bugiri to the south-west, Butaleja to the north-west and the Republic of Kenya to the east. The location, farming system, climate and vegetation of the study area have been previously described [18-20]. Cattle are the main tsetse hosts in Tororo district [11,12] contributing up to 54% of all tsetse blood meals with the rest taken from pigs and monitor lizards (*Varanus niloticus*) [12]. *Glossina fuscipes fuscipes* and *G. pallidipes* are the main tsetse fly species in the district [12]. At the time of the study, the district had estimated cattle population of 37,345 in 401 villages (average = 93 cattle/village) [21]. There was no evidence of any mass treatment of cattle against AAT in Tororo District between 2010–2011.

### Study design and sampling methods

A cross sectional study was carried out involving taking of blood samples from all cattle in 57 of the 401 cattle containing villages of Tororo District. The cluster sampling methodology [22,23] implemented in *C Survey* version 2.0 [24] was used to determine the minimum number of clusters needed. Cluster sampling allows variation in the number of clusters and cattle that are sampled (e.g., some cattle in all clusters, all cattle in all clusters; all cattle in some clusters) to achieve the specified parameters. Owing to the large size of this survey, village selection was done by simple random sampling to reduce sampling errors that often result from inherent variability between different samples drawn from a large population (sampling frame). The sampling frame; comprising a complete list of all villages and their geo-referenced positions in Tororo District was obtained from the Coordinating Office for Control of Trypanosomiasis in Uganda (COCTU) and verified for completeness at Tororo District lands and planning offices.

Sample size was determined assuming a mean cattle population of 93 cattle per village [21], anticipated prevalence of AAT of 30% based on the experience of the investigators, and some published general trypanosome studies [25], the precision of the sample estimate (one half-length of the 95% confidence interval) of 5 percentage points and an intracluster correlation coefficient (ICC) of 0.15. The ICC estimate was based on reported rates of homogeneity (rho) for trypanosomiasis prevalence, noting high variability [26,27]. The number of cattle expected to be sampled per cluster was taken as the mean of the number of cattle per village in Tororo District (93). In total, fifty seven (57) clusters (villages) were needed to achieve the set level of precision for trypanosome prevalence estimation. The number of clusters selected fulfil the sampling assumption that the cluster means are normally distributed [28] indicating that a minimum of 30 clusters could have been used.

Infectious diseases (both vector-borne and non-vector-borne) display heterogeneity within a population; that is, they tend to cluster into epidemiological units; a group of animals that is of epidemiological significance in terms of the transmission and maintenance of infection, and therefore of disease control. For this reason, the sampling units/clusters for this study are villages; the epidemiological units for trypanosomiasis. A livestock containing village in Tororo District is here defined as a cluster because, in communal husbandry obtaining this is the epidemiological unit.

### Cattle blood sample collection

About 125 μl of blood was collected from the middle ear vein and applied onto a designated sample area of the classic Flinders Technology Associates (FTA®) cards (Whatman Bioscience, Cambridge, UK), avoiding cross contamination [29,30]. Blood samples were then allowed to air-dry, labelled with village name, parish, sub county, county and date of collection. They were packed in foil pouches with a silica gel desiccant (Sigma Aldrich, Co., Life sciences, USA) prior to shipping to the University of Edinburgh, UK for analysis.

### DNA extraction

DNA was extracted and eluted in Chelex®100 resin (Sigma Aldrich, Co., Life sciences, USA) from five 3 mm FTA sample discs according to a previously described protocol [30,31]. Eluted DNA samples were kept at −20°C for long term PCR analyses or 4°C if they were to be analysed within a few days after extraction.

### Trypanosome DNA detection

Eluted DNA samples were screened for different trypanosome species using a single pair of primers (CR and BR) previously designed to amplify internal transcribed spacer (ITS1) of trypanosome ribosomal deoxyribonucleic acid (rDNA) and thermo cycling conditions as previously described [32]. The ITS1- PCR was carried out in a 25 μl reaction volume; 20 μl of which was the PCR master mix and either 5 μl of the test sample or negative control eluate or positive control DNA. The master mix was made of 10x-reaction buffer (670 mM Tris–HCl pH 8.8, 166 μM (NH4)_2_SO_4_, 4.5% Triton X-100, 2 mg/ml gelatin) (Fisher Biotech), 1.0 mM MgCl_2_, 200 μM of each dNTP, 5 μM each of the CF and BR primers, 0.5U of *Taq* DNA polymerase (Fisher Biotech) and 15.2 μl RNase-free molecular grade water.

To determine which samples were infected with either *T. brucei* or *T. b. rhodesiense*, multiplex PCR [33] was carried out on each of the samples from which a 450 bp fragment was detected on ITS1-PCR. Multiplex PCR was carried out in 25 μl reactions using primers and conditions as previously described [33]

To determine the commonest *T.congolense* genotype circulating in Tororo District, all samples from which a ≥600 bp fragment was amplified on ITS1-PCR were initially tested for *T.congolense savannah* using a single pair of primers (TCS1 & TCS2) and thermo cycling conditions as previously described [34]. All samples that were positive for *T.congolense* DNA on ITS1-PCR were positive for *T.congolense savannah.* For this reason, no more *T.congolense* genotype-specific (kilifi, tsavo, forest) PCRs were performed although a few co-infections with different *T.congolense* genotypes could have been possible. The PCR was carried out in a 25 μl reaction volume; 20 μl of which was the PCR master mix and either 5 μl of the test sample or negative control eluate or positive control DNA. The master mix was made of 10x-reaction buffer (670 mM Tris–HCl pH 8.8, 166 μM(NH4)_2_SO_4_, 4.5% Triton X-100, 2 mg/ml gelatin) (Fisher Biotech),, 4.5% Triton X-100, 2 mg/ml gelatin) (Fisher Biotech), 0.75 mM MgCl_2_, 200 μM of each dNTP, 12.5 μM each of the TCS1 & TCS2 primers, 1U of *Taq* DNA polymerase (Fisher Biotech) and 13.05 μl of RNase-free water.

PCR products for the three sets of PCRs were separated in 1.5% agarose (Bio Tolls Inc. Japan), stained in GelRed™ (Biotium, Inc., USA) and visualised on an ultraviolet transilluminator for fragment size determination

### Statistical analysis

Apparent prevalences and their confidence intervals were estimated using the generalized estimating equation models to adjust for correlations within communities. True prevalences were calculated using the Rogen-Gladen estimator. Intra cluster correlations were estimated using a 1-way mixed effect ANOVA model. All statistical analyses were performed using the R statistical software version 3.0.2. ArcMap v10.3 was used to map prevalence estimates in different villages.

### Ethical clearance

This study was reviewed by the Makerere University College of Veterinary Medicine Animal Resources and Biosecurity ethical review board for compliance to Animal use and Care standards. It was then forwarded to the Uganda National Council for Science and Technology and approved under approval number HS1336.

## Results

### Demographic characteristics of the study population

Six thousand fifty four blood samples were taken from all cattle in 57 villages in Tororo District. The mean number of cattle per village was 106 (4–232). The mean number of cattle per household was 4. The demographic characteristics of the study population are summarized in Table 1. Almost all the animals belonged to the Boran × short horn Zebu cross-breed. Approximately half of the population were female.

**Table 1:** **Description of the animal population**

**Study population attributes**	**Attribute level (N = 6054)**	
	**Sampled (n)**	**%**
**a) Age**		
0-12 months	1205	19.9
13-24months	1264	20.9
25-36months	953	15.7
>36 months	2632	43.5
**b) Sex**		
Female	3117	51.5
Male	2568	42.4
Neutered	369	6.1
**c) Breed**		
Boran × short horn Zebu cross	5869	96.9
Boran × Holstein Friesian cross	89	1.5
African short horn Zebu (Nkedi)	96	1.6

### Bovine trypanosome species prevalence

Out of 6,054 cattle sampled, 850 were infected with a single trypanosome species and a further 78 animals had mixed trypanosome infections (Table 2). The most common co-infections observed were *T. vivax* and *T. b. brucei* (37 animals) and *T. vivax* and *T. congolense* (34 animals). The overall prevalence of different trypanosome species was 15.3% (95% CI; 12.2-19.1%). *T.brucei s.l.* constituted the smallest proportion of trypanosomes in cattle in Tororo District.

**Table 2:** **Prevalence of different trypanosome species in Tororo district (September- November 2011)**

**Trypanosome species**	**Positive/n**	**Apparent prevalence** ^**a**^ **(95%CI)**	**% True prevalence** ^**b**^	**% Herd prevalence** ^**c**^	**ICC** ^**d**^
Overall	928/6048	15.3 (12.2-19.1)	-	91	0.11
*T. vivax*	813/6053	13.4 (10.6-16.8)	17.4	91	0.09
*T. congolense savannah*	127/6049	2.1 (1.4-3.1)	2.3	53	0.04
*T. b. brucei*	69/6050	1.1 (0.7-1.8)	1.2	35	0.02
*T. b. rhodesiense*	2/6050	0.03 (0.0-0.1)	0.03	04	0.00

^*a*^Adjusted for intra cluster correlation using generalised estimating equation (GEE) model.

^b^Rogan-Gladon estimator assuming 100% specificity and sensitivities of 77.4%, 90.9%, 95%, 95% for *T.vivax, T. c. savannah, T. b. brucei* and *T. b. rhodesiense* [32], respectively.

^c^Due to the local farming systems, all animals within a certain village are considered as a herd.

^d^Intra cluster correlation coefficient or rate of homogeneity (rho).

### Herd level prevalence of different bovine trypanosome species

There was a very large variation in the prevalence of different trypanosome species between different villages (clusters) with some villages recording 0 infection rates while others recording very high infection rates of up to 43% (Table 3). As a result, the rate of homogeneity (roh)/ intra-cluster correlation coefficient (ICC) for any trypanosome infection was estimated at 0.11. The most common trypanosome species was *T. vivax* with an apparent prevalence of 13.4 (10.6-16.8) occurring in 52 out of 57 communities respectively. *T. c. savannah* and *T.brucei s.l.* were detected in 53% and 35% of all villages sampled. *T.b. rhodesiense* was detected only in 2 of the 57 (3.5%) villages sampled.

**Table 3:** **Herd level prevalence of different trypanosome species in Tororo district**

**Village**	**Sampled (n)**	**All trypanosomes**	***T. vivax***	***T.c.savannah***	***T.brucei s.l.***	***T.b rhodesiense***
Adumai	124	0.8	0.8	0.0	0.0	0.0
Akadoti	60	20.0	16.7	3.3	0.0	0.0
Alupe_A	66	1.5	1.5	0.0	0.0	0.0
Alupe_B	60	36.7	35.0	0.0	3.3	0.0
Agolol	163	8.6	8.6	0.0	0.0	0.0
Asinge-C	232	0.4	0.4	0.0	0.0	0.0
Atapara-Kaleu	160	20.6	18.1	1.9	1.2	0.0
Biranga-B	82	1.2	1.2	0.0	0.0	0.0
Biranga-A	20	35.0	25.0	10.0	5.0	0.0
Chawolo_ A	213	11.7	11.7	0.0	0.0	0.0
Chawolo_B	188	15.4	11.7	2.7	1.6	0.5
Dida	100	33.3	34.0	1.0	3.1	0.0
East-Central	56	26.8	25.0	1.8	0.0	0.0
Iyopoki	86	1.2	1.2	0.0	0.0	0.0
Iyoriang	118	11.9	11.0	0.0	1.7	0.0
Kadanya	132	3.8	3.8	0.0	0.0	0.8
Kajalau	64	28.1	21.9	7.8	3.1	0.0
Kasoli	197	19.8	19.8	0.0	0.0	0.0
Katandi	106	13.2	12.3	0.9	0.0	0.0
Kateki	69	4.3	4.3	0.0	0.0	0.0
Kirewa	132	22.7	22.7	0.8	7.6	0.0
Kisera	101	5.9	5.9	0.0	0.0	0.0
Kogala	127	3.1	2.4	0.8	0.0	0.0
Komolo	112	0.0	0.0	0.0	0.0	0.0
Macharimeri	180	38.9	36.7	2.8	4.4	0.0
Mailombili	50	20.0	16.0	4.0	0.0	0.0
Maliri	71	22.5	15.5	2.8	4.2	0.0
Mella	144	0.7	0.7	0.0	0.0	0.0
Mikwana	169	21.3	19.5	5.3	0.0	0.0
Munyinyi	164	33.7	26.4	8.5	3.0	0.0
Mwelo	36	38.9	36.1	11.1	0.0	0.0
Ngeta-A	127	18.9	18.1	0.8	0.0	0.0
Nyabanja	139	30.9	23.0	9.4	2.9	0.0
Nyafumba	94	18.1	16.0	2.1	0.0	0.0
Nyemera	88	11.4	10.2	1.1	1.1	0.0
Okwira	18	33.3	33.3	0.0	0.0	0.0
Opule	72	9.7	9.7	0.0	0.0	0.0
Oriyoyi	124	26.6	26.6	0.0	0.0	0.0
Osia	112	0.0	0.0	0.0	0.0	0.0
Pabasi	110	2.7	2.7	0.0	0.0	0.0
Pabendo	76	28.9	26.3	3.9	0.0	0.0
Pamaraka	80	18.8	11.2	8.8	0.0	0.0
Panyandere	74	1.4	1.4	0.0	0.0	0.0
Pasaya	104	21.4	16.3	7.8	0.0	0.0
Pawira	215	5.1	2.8	1.4	0.9	0.0
Poti	78	6.4	6.4	0.0	0.0	0.0
Rubuleri	91	31.9	29.7	6.6	4.4	0.0
Rukuli	32	9.4	9.4	0.0	0.0	0.0
Segero	100	20.0	17.0	3.0	2.0	0.0
Seseme	52	28.8	25.0	0.0	7.7	0.0
Sesera	49	42.9	24.5	18.4	4.1	0.0
Singisi	76	26.3	17.1	10.5	0.0	0.0
Ticaf	204	0.0	0.0	0.0	0.0	0.0
Totokidwe	144	25.7	24.3	1.4	2.1	0.0
Tuba	90	0.0	0.0	0.0	0.0	0.0
Wakasiki	119	29.4	22.7	2.5	5.0	0.0
West-Central	4	0.0	0.0	0.0	0.0	0.0

### Prevalence of *T.brucei s.l*

**Figure 1:**
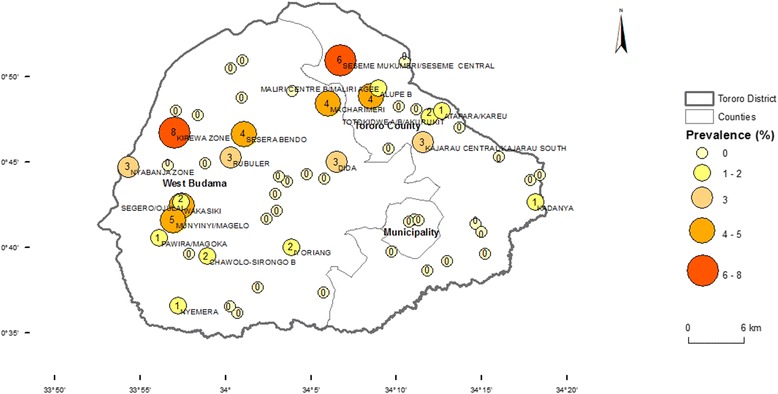
**Prevalence of** ***T.brucei s.l.*** **in cattle in 57 villages of Tororo District, Uganda.** *T.brucei s.l.* prevalence was categorized into five classes for which symbols differ in size and colour. County boundaries were included for ease of assessment of the location of sample sites within the district. The estimated number of cases per 100 animals is presented within the symbols. Only names of villages with the highest prevalence estimates are added.

Only 69 blood samples were positive for *T.brucei s.l.* (Figure 1)*.* Further characterisation by multiplex PCR identified 2 of the 69 samples as positive for the human infective *T. b. rhodesiense.* The two *T. b. rhodesiense* positive samples were from the villages of Chawolo-Sironga B and Kadanya; one positive sample from each of the two villages

### Spatial distribution of bovine trypanosomes

**Figure 2:**
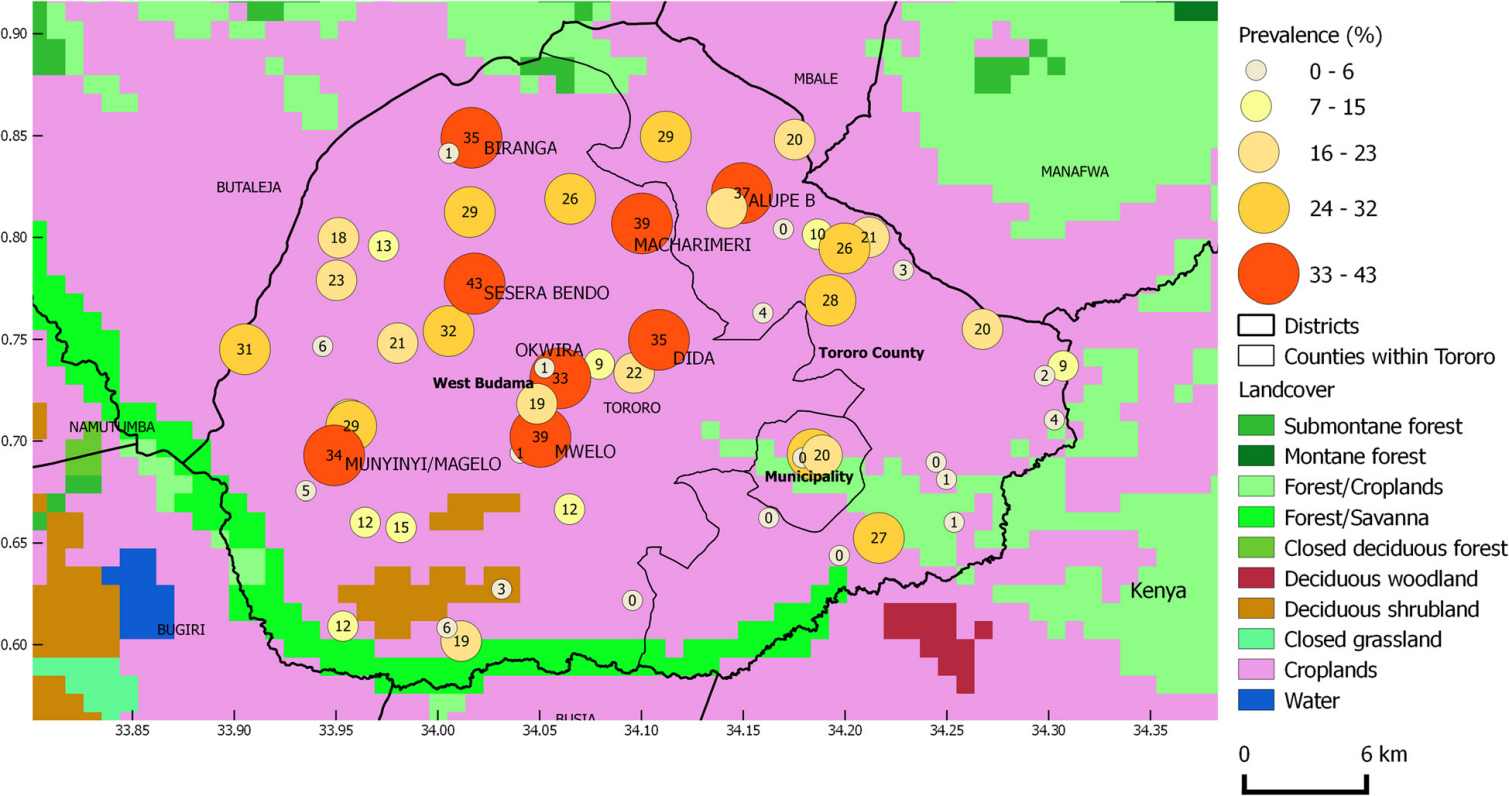
**Spatial distribution of bovine trypanosome species and land cover.** The overall prevalence of different trypanosome species in each village was categorized into five classes for which symbols differ in size and colour. County boundaries were included for ease of assessment of the location of sample sites within the district. The estimated number of cattle infected with different trypanosome species per 100 animals are presented within the symbols. Only name labels of villages with the highest prevalence estimates were added to avoid overcrowding the map. A background layer of land cover classes (GLC2000) was added to assess the likely effect of land use on trypanosome prevalence.

The lowest prevalences were registered along the Kenyan Border while the highest prevalences were recorded in villages in the northern and the western parts of the district bordering the districts of Manafwa and Mbale (Figure 2).

## Discussion

To determine the burden and spatial distribution of African trypanosomes in crop-livestock production systems in Tororo District, south-eastern Uganda, cattle blood samples were taken and tested for different trypanosomes between September and December 2011. Six thousand and fifty four cattle blood samples from 57 villages were analysed for different trypanosomes. The overall prevalence for different trypanosomes in Tororo District of 15.3% (95% CI; 12.2-19.1%) was comparable to those previously reported in this region [12,35]. Individual species prevalences were higher than those reported in previous studies [12,35]. The most pathogenic species for cattle, *T. vivax* and *T. congolense* were recorded at 13.4% (95% CI; 10.6-16.8) and 2.1% (95% CI; 1.4-3.1) respectively. *T. brucei* s.l was observed at a prevalence of 1.1% (95% CI; 0.7-1.8) with a low frequency [36] with 2 of 69 positive samples being positive for *SRA* gene, indicative of human infectivity [10].

The prevalence of different trypanosome species greatly varied between different villages ranging from 0-43% (Table 3). Villages with medium to high AAT prevalence were clustered along the northern and western borders of Tororo District along the Kenyan border (Figure 2). These areas are mainly covered by forest/savannah vegetation and croplands interspersed with cattle which make the ideal conditions for tsetse infestation. Trypanosome prevalence and land use showed a positive spatial association, which might explain the high prevalences found in the north and western boarders of Kenya (Figure 2). This could be as a result of the differences in tsetse apparent density and veterinary care between villages [37].

*T. vivax* was detected in 91% of all sampled villages. *Glossina fuscipes fuscipes* has been reported to be the commonest tsetse fly species in eastern Uganda [38-40] and was the commonest tsetse species caught in a recent survey at 161 locations in Tororo District [37]. *G. f. fuscipes* is a better vector for *T.vivax* than *G. pallidipes* [37,40], which is scarce despite recent re-invasion in this part of Uganda [35]. This aside, mechanical transmission may explain why *T. vivax* was detected in most sampled villages. *T. vivax* undergoes a short life cycle in the proboscis of tsetse [42-44] and a fast build-up of parasitaemia in mammalian hosts [45] which may contribute to *T.vivax* detection in cattle.

Prevalences of *T. congolense, T.b. brucei and T. b. rhodesiense* in Tororo District were lower than observed for *T. vivax*. However, *T. c. savannah* was detected in slightly above half (53%) of all the herds (villages) screened. The wider distribution of *T. c. savannah* in Tororo District herds may be as a result of re-invasion of *G.pallidipes* in Tororo, associated with risk of transmission of *T. c. savannah* [38].

*T. congolense* and the haemorrhagic strain of *T.vivax* are the most pathogenic trypanosome species of cattle in East Africa causing an acute and fatal disease compared to a more chronic form of AAT caused by other bovine trypanosome species and strains [34,46]. That *T. congolense* infections progress to a fatal disease rapidly, demanding treatment, may explain why *T. congolense* was detected in low prevalences in apparently healthy animals screened in the 57 villages of Tororo [47]. The very high prevalence and wide distribution of *T. congolense* and *T.vivax* in Tororo District indicates that these highly pathogenic trypanosome species continue to constrain livestock production calling for sustainable trypanosomiasis control measures.

*T. b brucei* and *T. b. rhodesiense* were detected in low individual species and herd prevalences. The very low prevalence (0.03%) of *T. b. rhodesiense* in cattle indicates that there still remains a low risk of transmission of *T. b. rhodesiense* from cattle to humans. This is especially so in the two villages of Chawolo-Sironga B and Kadanya where two cattle blood samples were found positive for T*. b. rhodesiense*, one sample from each village. Given that ITS1-PCR has slightly lower sensitivity (95%) to *T.brucei s.l.* than the species specific PCR (~100%) *T.brucei s.l.* may have been under detected by ITS1-PCR [48-50].

The last outbreak of sleeping sickness in Tororo District was in 2000/2001 [50]. Since then, there have not been reports of the human disease in the district. That *T. b. rhodesiense* was detected only in 2 out of 6,054 cattle sampled shows that the cattle reservoir for this infection in Tororo District is persistent and is of concern. The presence of human infective *T. b. rhodesiense* animal carriers within isolated villages in Tororo District indicates that humans in these isolated villages remain at risk of acquiring infection given the abundance of tsetse flies [51].

Cattle are grazed communally in large herds (average of 106) in Tororo but at night are kept in smaller groups (average of 4) in night-shades or tethered around homesteads [19,20,37]. The number of animals per household is low typical of livestock holding in south-eastern Uganda. Boran and African short horn Zebu (Nkedi) hybrids and Nkedi are the major cattle breeds kept in Tororo District [19,20,52]. The male to female cattle ratio is high (0.8) in Tororo, since farmers need to retain bulls (whole or neutered) for 3 years or more to provide draught power in these mixed crop-livestock systems [19,53]. Cattle over three years of age have been associated with a higher risk of infection and spread of human infective *T.b rhodesiense* [54]. Production systems that retain a very high proportion of cattle above 3 years of age pose a risk for zoonotic *T.b. rhodesiense* HAT transmission. Improving livestock health by controlling tsetse and trypanosomiasis will reduce HAT incidence, enhance livestock production and livestock-crop integration thereby reducing poverty and hunger [17,55-57]

The individual and herd prevalences reported in this study show that African trypanosomiasis remains a constraint to livestock production and livestock-crop integration in Tororo District despite farmer and local government-led tsetse and trypanosomiasis control efforts [36]. Lack of sustainability caused by insufficient follow-up, civil unrest and inadequate financing have resulted in failure of most tsetse control programs in Uganda [58]. Cattle as persistent reservoirs of human HAT should be treated to remove risk to poor communities in affected districts [6]. To prevent reinfection by tsetse control, the control methods used ought to be effective and sustainable. This requires that such methods are tailored to the limited budgets of poor rural livestock keepers and are effective against multiple endemic livestock diseases [59]. Control methods based on the use of restricted application of insecticides (RAP) on predilection sites for tsetse and/or ticks or spraying larger animals could serve to reduce the cost of RAP and offer added value of targeting ticks and biting flies making it easily adoptable for routine tsetse control [60-62].

## Conclusion

African animal trypanosomiasis continues to be one of the main constraints to livestock production in Uganda. The current study indicates that the prevalence of different trypanosome species in Tororo District is still high despite government and farmer-led tsetse and trypanosomiasis control efforts. There is need to further intensify tsetse and trypanosomiasis control efforts in the district preferably by employing effective and sustainable tsetse and trypanosomiasis control methods. Such methods would need to be compatible to inelastic budgets of resource poor livestock keepers and be effective against most important endemic vector-borne diseases namely; tsetse and tick-borne diseases.
